# LncSIK1 enhanced the sensitivity of AML cells to retinoic acid by the E2F1/autophagy pathway

**DOI:** 10.1111/cpr.13185

**Published:** 2022-01-29

**Authors:** Ke Wang, Jun‐da Liu, Ge Deng, Zi‐yao Ou, Shu‐fang Li, Xiao‐ling Xu, Mei‐Ju Zhang, Xiao‐Qing Peng, Fei‐hu Chen

**Affiliations:** ^1^ 12485 School of Pharmacy Anhui Medical University Hefei China; ^2^ Inflammation and Immune Mediated Diseases Laboratory of Anhui Province Anhui Institute of Innovative Drugs Hefei China; ^3^ 12485 Anhui Province Key Laboratory of Major Autoimmune Diseases Anhui Medical University Hefei China; ^4^ Department of Anesthesiology the First Affiliated Hospital of Anhui Medical University Hefei China; ^5^ Department of Obstetrics and Gynecology the First Affiliated Hospital of Anhui Medical University Hefei China

**Keywords:** acute myeloid leukaemia, autophagy, E2F1, LncSIK1, retinoic acid

## Abstract

**Objectives:**

This study aimed to investigate the biological impacts and possible mechanisms of a novel lncRNA, LncSIK1, in AML progression and retinoic acid‐regulated AML cell development.

**Materials and Methods:**

The expression pattern of LncSIK1 was evaluated by qPCR and fluorescence in situ hybridization. CCK‐8 assay, immunofluorescence, Wright‐Giemsa staining, flow cytometry and Western blotting were performed to assess cell proliferation and differentiation. Bioluminescence imaging and H&E staining were used to detect AML progression *in vivo*. RNA or chromatin immunoprecipitation assays were conducted to measure the interaction of E2F1 and LncSIK1 or the LC3 and DRAM promoters. Autophagy was measured by transmission electron microscopy and Western blotting.

**Results:**

LncSIK1 was silenced in bone marrow mononuclear cells from AML patients compared with those from healthy donors. LncSIK1 strengthened the effect of retinoic acid in inducing cell differentiation and inhibiting cell proliferation in AML cells. Moreover, the silencing of LncSIK1 was critical to maintaining AML leukaemogenesis, as LncSIK1 enhancement retarded AML progression *in vivo*. Mechanistically, in NB4 cells, LncSIK1 recruited the E2F1 protein to the promoters of LC3 and DRAM and induced autophagy‐dependent degradation of the oncoprotein PML‐RARa. However, LncSIK1 blocked E2F1 expression and the E2F1‐mediated transcription of LC3 and DRAM, thereby relieving aggressive autophagy in Molm13 cells.

**Conclusions:**

Taken together, these data indicated that LncSIK1 was an important regulator of AML development through regulating the E2F1/autophagy signalling pathway.

## Graphical abstract

The hypothetical regulatory network of LncSIK1 in NB4 and Molm13 cells.

1. LncSIK1 exerted its significant positive effect on the sensitivity of AML cells to retinoic acid.
2. LncSIK1 enhancement retarded AML progression *in vitro* and *in vivo*.
3. The E2F1/autophagy axis played an important role in LncSIK1‐regulated AML development.

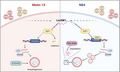

## 1 INTRODUCTION

Acute myeloid leukaemia (AML) is an invasive haematopoietic malignancy with highlight genetic and epigenetic heterogeneity.^1^ Even though standard therapy options are available for AML patients, AML is still a leading cause of cancer‐related death due to recurrence and drug resistance. According to the GLOBOCAN 2020 database of the World Health Organization International Agency for Research on Cancer, leukaemias account for 3.1% of global cancer‐related mortality, with approximately 310 000 deaths worldwide. The imbalance between aggressive self‐renewal and blocked differentiation of immature myeloid precursors is a crucial factor in the accumulation of leukaemic blasts and bone marrow failure.^2^, ^3^ Therefore, revising the imbalance by inducing differentiation of immature myeloid precursors holds great promise for AML therapeutic strategy. Recently, researchers have established a ncRNA atlas of the human haematopoietic landscape and identified a set of lncRNAs that are highly relevant to haematopoietic stem/progenitor cell (HSPC) differentiation or hijacked during leukaemic transformation.^4^ However, the regulatory roles and the underlying mechanisms of numerous lncRNAs in haematopoiesis and leukaemogenesis are not entirely understood. All‐trans retinoic acid (ATRA) is a classic clinical differentiation‐inducing agent for AML.^5^ ATPR is a novel ATRA derivative with substantial pharmacological activity on the differentiation of AML cells.^6^, ^7^ We previously screened, by microarray analysis on NB4 cells in the context of ATPR, a distinct set of lncRNAs that may be related to AML progression.^8^ In the present work, LncSIK1 (LncSIK1‐1:1; NONHSAT082403, NONCODE v4; Location: chr21:43390213–43390889) was identified as one of the lncRNAs induced during retinoic acid treatment.

Autophagy serves as a catabolic machine whereby bulk cytosol and organelles are sequestered in autophagosomes and delivered to lysosomes for degradation.^9^ The role of autophagy in AML, however, becomes complicated when considering heterogeneity. On the one hand, the induction of cell differentiation in AML is partially attributed to the activation of autophagy.^10^, ^11^ For instance, accelerated degradation of the promyelocytic gene‐retinoic acid receptor alpha (PML‐RARa) protein via the autophagosome‐lysosome pathway appears to be critical for enhancing the differentiation of AML.^12^ On the other hand, several specific oncogenes support aggressively high levels of basal autophagy in AML cells, for example, Fms‐like tyrosine kinase 3‐internal tandem duplication (FLT3‐ITD).^13^ Inhibition of FLT3 activity decreases autophagy in cultured cells and primary AML samples and subsequently alleviates leukaemic burden and resistance to FLT3 inhibitor in AML.^14^, ^15^ Despite the publication of these studies indicating the contradictory role of autophagy in AML, the detailed molecular mechanisms of the role are still poorly understood. Some researchers have suggested that lncRNAs are involved in carcinogenesis by enhancing or suppressing autophagy.^12^, ^16^, ^17^ Therefore, the potential role of the lncRNA/autophagy axis in AML cell biology as a mechanism of AML progression is particularly worthy of elucidation.

This study investigated the biological impacts and possible mechanisms of LncSIK1 in AML progression and retinoic acid‐regulated AML cell development. Two AML cell lines (NB4 and Molm13, which are hypersensitive or hyposensitive to ATRA‐ or ATPR‐induced cell differentiation) were used for this study.

## 2 MATERIALS AND METHODS

### 2.1 Human samples and cell isolation

Primary AML leukaemia cells were isolated from the bone marrow of AML patients at diagnosis from the Second Hospital of Anhui Medical University. Briefly, bone marrow blood was slowly layered over Ficoll‐Paque PLUS solution (GE Healthcare Life Sciences, Sweden) and centrifuged at 550×*g* for 25 mins. The mononuclear cells in the interphase layer were carefully transferred into another fresh tube, washed with Hank's balanced salt solution (Beyotime Biotechnology) twice and then applied to subsequent experiments. CD34^+^ haematopoietic stem/progenitor cells (HSPCs) and CD34− cells were purified from the cord blood of healthy donors from the First Affiliated Hospital of Anhui Medical University using Ficoll‐Paque PLUS and anti‐CD34‐coated magnetic beads (Miltenyi Biotec). Briefly, we isolated mononuclear cells from fresh cord blood as described above. Next, a single cell suspension obtained was incubated with anti‐CD34‐coated beads and selected on autoMACS Pro (Miltenyi Biotec). CD34^+^ cells were identified by incubation with CD34 antibody (BioLegend, USA) and analysis with flow cytometry (CytoFLEX, Beckman Coulter).

### 2.2 Xenotransplantation experiments

NOD/ShiLtJGpt‐Prkdc^em26Cd52^Il2rg^em26Cd22^/Gpt (NCG) immunodeficient mice (animal certificate number: 202011417) were obtained from Gempharmatech (Nanjing, China), and NOD‐Prkdc^scid^Il2rg^em1^/Smoc (M‐NSG) immunodeficient mice (animal certificate number: 20170010009479) were obtained from the Shanghai Model Organisms Center (Shanghai, China). Male mice aged of 6–9 weeks were used for the experiments. All animals were fed under a specific pathogen‐free facility (temperature: 23–24°C, humidity: 40%–45%) in the Laboratory Animal Center of Anhui Medical University. All experiments on animals were performed in accordance with the institutional ethical guidelines for animal experiments (Ethics Number: LISC20190751).

For the subcutaneous tumour xenograft study procedure (Figure S1A), selected LncSIK1‐WT or EV NB4 cells were diluted to a concentration of 5 × 10^7^ cells/ml in phosphate buffer saline (PBS) (HyClone). NCG mice were subcutaneously injected with 0.1 ml of the suspension (5 × 10^6^ cells) into either side of the flank area. Mice were sacrificed 2 weeks later, and tumours were weighed, fixed and subjected to haematoxylin and eosin (H&E) staining.

For the intravenous bone marrow engraftment study procedure (Figure S1B), M‐NSG mice were acclimated for 2 weeks before pretreatment with 150 mg/kg cyclophosphamide delivered intraperitoneally once a day for 2 days. After a 24‐h rest period, selected LncSIK1‐WT or EV Molm13 cells were diluted to a concentration of 5 × 10^7^ cells/mL in PBS and M‐NSG mice were injected with 0.1 ml of the suspension (5 × 10^6^ cells) into the tail vein. Three weeks after inoculation, the mice were sacrificed for analysis. Bioluminescence imaging (BLI) was applied to evaluate human cell infiltration (BLI‐positive signal) in mice. H&E staining and flow cytometry were employed to detect cell engraftment in bone marrow, peripheral blood, liver, kidney and spleen as described.

### 2.3 Statistical analysis

Data were assessed using the software SPSS 19.0 (SPSS). Data with a normal distribution were analysed by Student's *t* test, while data with a non‐normal distribution were analysed by the Mann‐Whitney nonparametric test. Comparisons between groups were performed using one‐way ANOVA followed by Tukey's multiple comparison. Correlations between the samples were evaluated statistically through Pearson's correlation coefficient. Error bars depict the mean ± standard error of the mean (SEM). P values of less than.05 were considered as statistically significant.

Further details of the materials and methods are included in Additional file S1.

## 3 RESULTS

### 3.1 Characterization of LncSIK1

**FIGURE 1:**
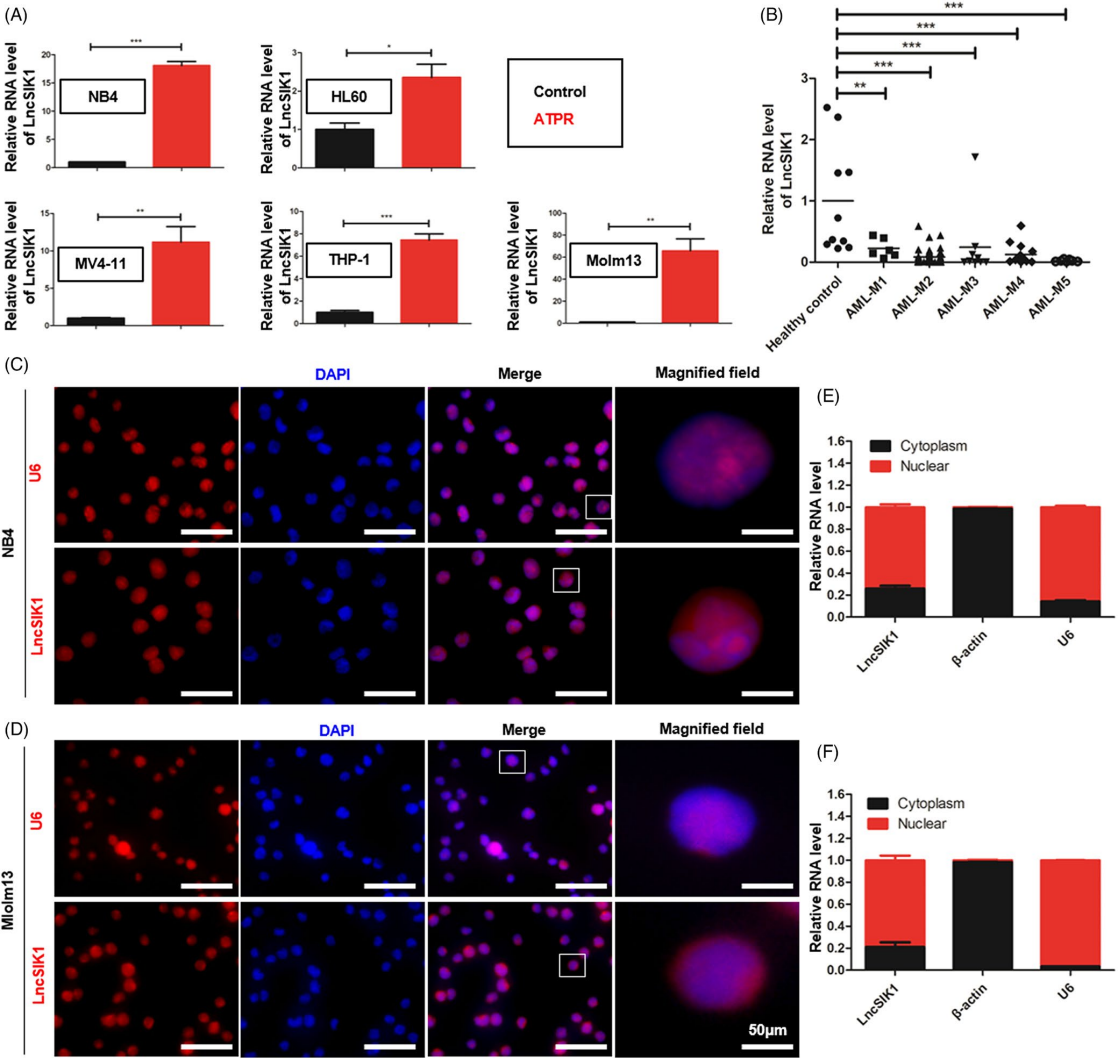
Characterization of LncSIK1. (A) Relative expression of LncSIK1 in AML cell lines in the absence or presence of ATPR, as detected by qPCR. (B) Relative expression of LncSIK1 in BMNCs from AML primary patients (AML M1‐M5 subtype) compared with BMNCs from healthy donors (control), as detected by qPCR. AML‐M1 (*N* = 6), AML‐M2 (N = 42), AML‐M3 (*N* = 9), AML‐M4 (*N* = 15), AML‐M5 (*N* = 7) and healthy controls (*N* = 10). Error bars depict mean ± SEM. (C and D) FISH analysis was performed to determine the location of LncSIK1 in NB4 (C) and Molm13 (D) cells. (E and F) LncSIK1 distribution in NB4 (E) and Molm13 (F) cells was analysed by cytoplasmic and nuclear fractionation assay and qPCR. β‐actin served as a loading control and a positive control for cytoplasmic gene expression. U6 served as a positive control for nuclear gene expression. Bar, 50 μm. Three independent technical replicates (from three biological replicates) were performed and included in the analysis. ns: no significance, *p < 0.05; **p < 0.01; ***p < 0.001

To systematically identify lncRNAs in the differentiation system of AML cells, we analysed microarray analysis data from NB4 cells with or without ATPR treatment.^8^ We focussed our research on LncSIK1, which was upregulated after ATPR treatment. To confirm the upregulation of LncSIK1 induced by ATPR, the LncSIK1 levels of five AML cell lines (NB4, HL60, Molm13, THP‐1 and MV411) after ATPR treatment were measured by qPCR. As expected, the mRNA expression of LncSIK1 was increased in the ATPR group compared with controls in all cell lines (Figure 1A). The LncSIK1 expression pattern in the BMNCs of AML patients at diagnosis or healthy donors was further determined. Primary patients with AML were classified into AML‐M1 (*n* = 6), AML‐M2 (*n* = 42), AML‐M3 (*n* = 9), AML‐M4 (*n* = 15) and AML‐M5 (*n* = 7) according to the French‐American‐British classification.^18^ The level of LncSIK1 in BMNCs of AML patients was notably lower than that of healthy controls (*n* = 10), suggesting that LncSIK1 may be implicated in the pathogenesis and progression of AML (Figure 1B). Because the functions of lncRNAs are closely related to their subcellular location, FISH analysis was carried out on AML cells to determine the location of LncSIK1. The results of NB4 (Figure 1C) and Molm13 (Figure 1D) cells illustrated that LncSIK1 resided in both the nuclear and cytoplasmic fractions. In addition, cytoplasmic and nuclear fractionation‐qPCR assays were performed in NB4 (Figure 1E) and Molm13 (Figure 1F) cells, and U6 and β‐actin were applied as positive controls in the nucleus and cytoplasm. In agreement with the results of FISH analysis, the qPCR results showed that LncSIK1 was located in both the nuclear and cytoplasm.

Thus, LncSIK1 was abnormally silent in AML and can be enhanced by retinoic acid.

### 3.2 LncSIK1 enhanced the sensitivity of AML cells to retinoic acid in vitro

**FIGURE 2:**
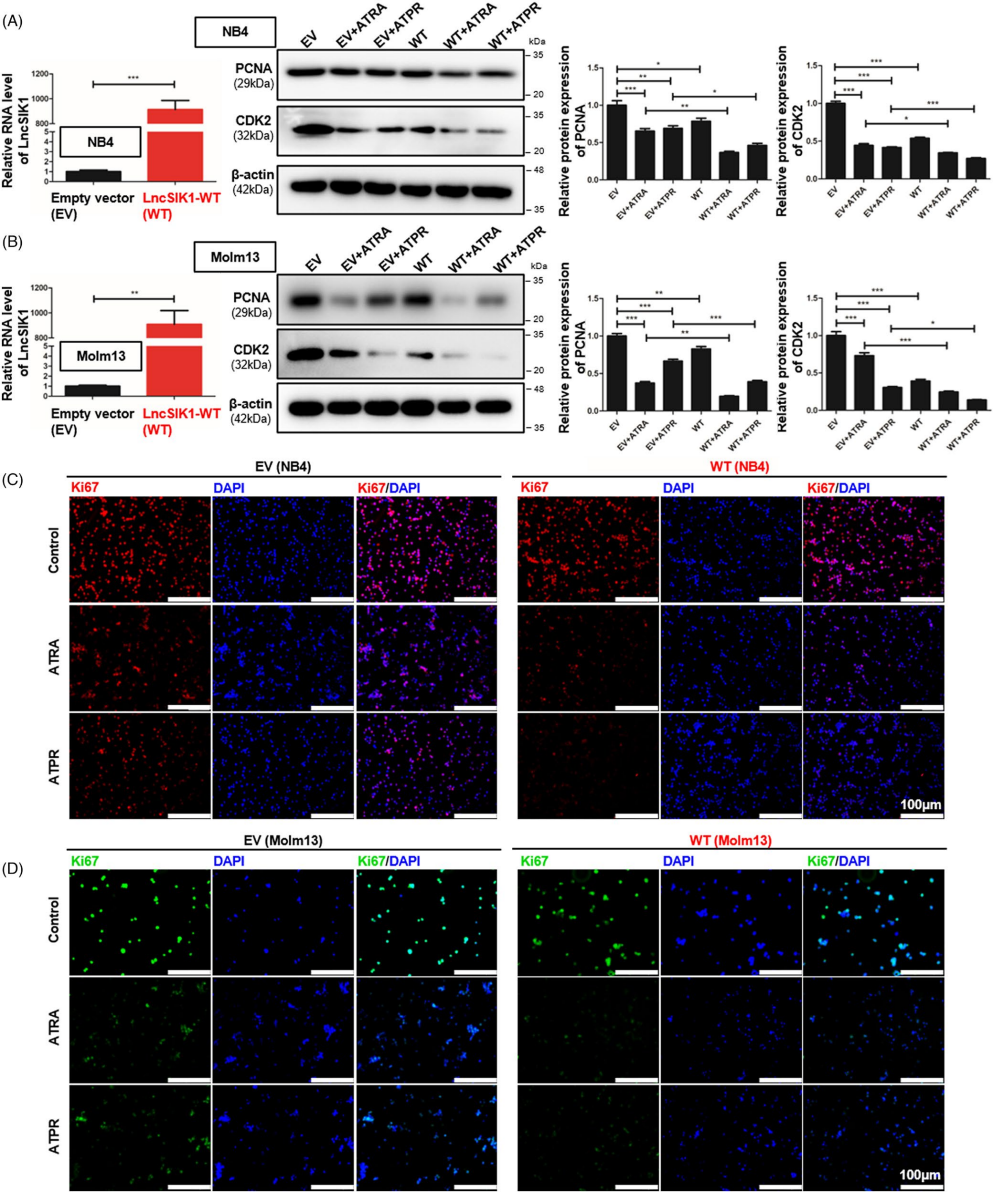
LncSIK1 enhanced retinoic acid‐induced reduction of cell proliferation in AML *in vitro*. NB4 and Molm13 cells were infected with lentiviral LncSIK1‐WT (WT) or empty vector (EV) plasmids and exposed to ATRA or ATPR (1 μM for 72 hours for NB4 cells, 10 μM for 72 h for Molm13 cells). (A and B) Effects of LncSIK1 enhancement on the protein expression of PCNA and CDK2 were analysed by Western blot. The overexpression efficiencies of LncSIK1 were confirmed by qPCR and are shown on the left (A, NB4 cells; B, Molm13 cells). (C and D) Immunofluorescence was used to detect the fluorescence intensity of Ki67 in NB4 (C) and Molm13 (D) cells after LncSIK1 enhancement. WT: cells infected with lentiviral LncSIK1‐WT plasmids. EV: cells infected with lentiviral empty vector plasmids. β‐actin served as a loading control. Bar, 100 μm. Three independent technical replicates (from three biological replicates) were performed and included in the analysis. ns: no significance, *p < 0.05; **p < 0.01; ***p < 0.001

**FIGURE 3:**
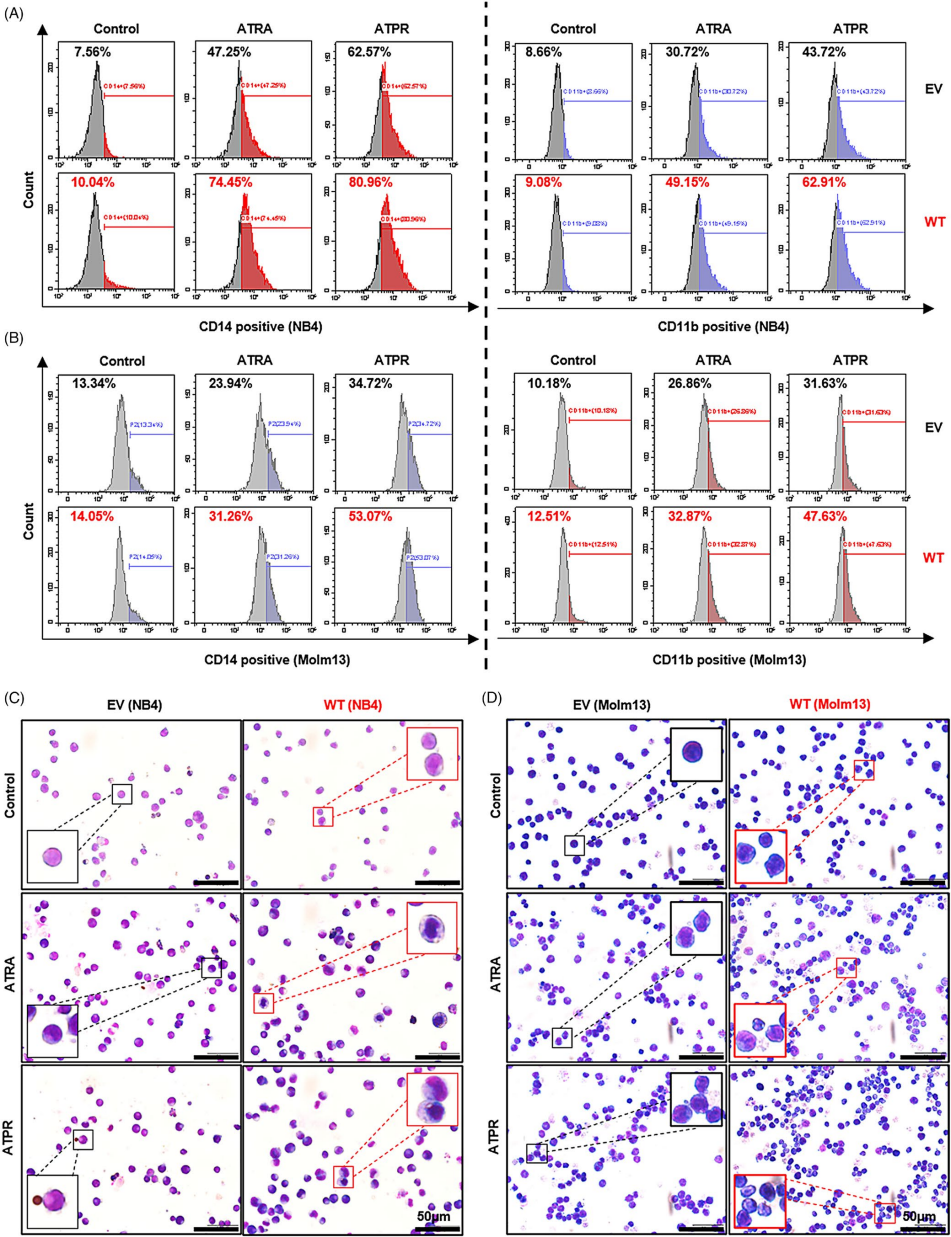
LncSIK1 enhanced retinoic acid‐induced cell differentiation in AML cells *in vitro*. NB4 and Molm13 cells were infected with lentiviral LncSIK1‐WT (WT) or empty vector (EV) plasmids and exposed to ATRA or ATPR (1 μM for 72 hours for NB4 cells, 10 μM for 72 hours for Molm13 cells). (A and B) The levels of the differentiation markers CD14 (left) and CD11b (right) were analysed by flow cytometry in NB4 (A) and Molm13 (B) cells. CD14 was applied as a marker for monocytic differentiation, and CD11b was applied as a marker for granulocytic differentiation. (C and D) Changes in the morphology of Wright‐Giemsa‐stained cells: (C) NB4 cells, (D) Molm13 cells. Purple: Nuclear. Blue: Cytoplasm. WT: cells infected with lentiviral LncSIK1‐WT plasmids. EV: cells infected with lentiviral empty vector plasmids. Bar, 50 μm

The specifically lower level of LncSIK1 in AML prompted us to investigate whether rescue of lncRNA could retard AML progression. NB4 and Molm13 cells were stably infected with pHBLV‐CMV‐LncSIK1‐EF1‐fLUC‐T2A‐PURO‐based lentiviral LncSIK1‐WT plasmids (WT) or empty vector (EV) followed by ATRA or ATPR incubation. Following infection, LncSIK1 levels were significantly enhanced (left, Figure 2A and B). As expected, LncSIK1 enhancement inhibited the expression levels of the cell proliferation marker PCNA and the cell cycle regulator CDK2 (right, Figure 2A and B). However, fluorescence microscopy results showed that no significant difference was observed in the fluorescence intensity of Ki67 after LncSIK1 enhancement, but LncSIK1 increased retinoic acid‐induced reduction of Ki67 expression in NB4 (Figure 2C) and Molm13 (Figure 2D) cells. Collectively, LncSIK1 has a profound effect on cell proliferation during retinoic acid treatment. Furthermore, despite the hypersensitivity of NB4 cells to retinoic acid, LncSIK1 enhancement still led to a drastic improvement in retinoic acid‐induced differentiation, as assessed by the fluorescence intensity of the specific monocytic marker CD14 (left, Figure 3A) and myeloid marker CD11b (right, Figure 3A). LncSIK1 significantly strengthened the retinoic acid‐induced differentiation of Molm13 cells (Figure 3B). NB4 (Figure 3C) and Molm13 (Figure 3D) cells after the indicated treatment were visually analysed by standard morphological criteria in Wright‐Giemsa‐stained samples. Both the indentation and bending of the nuclei and the decrease in the nuclear/cytoplasmic ratio indicated an increase in the differentiation of NB4 and Molm13 cells. In addition, retinoic acid induced significant cell shrinkage in Molm13 cells after LncSIK1 enhancement, indicating a potential role of LncSIK1 in cell death.

Together, the *in vitro* rescue studies demonstrated that enhanced expression of LncSIK1 substantially increased the responses of AML cells to retinoic acid in inhibiting proliferation and promoting differentiation.

### 3.3 The silencing of LncSIK1 was essential for maintaining the progression of AML in vivo

Of interest, our study shows that rescue of LncSIK1 alone does not regulate the cell development of AML. Therefore, we next determined whether the silencing of LncSIK1 is critical to maintaining the progression of AML. We generated two independent xenograft models using puromycin‐selected AML cells infected with LncSIK1‐WT or EV.^19^

**FIGURE 4:**
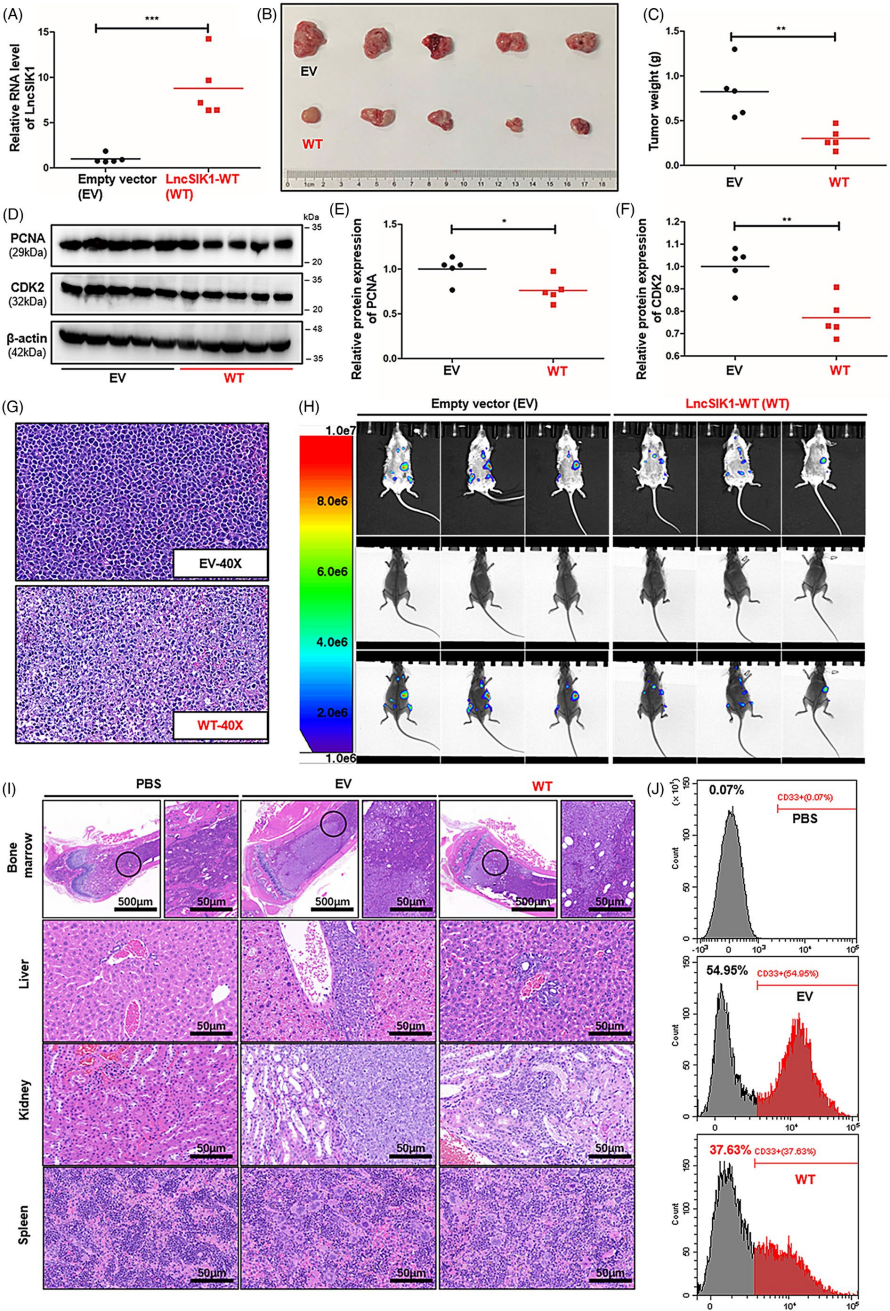
Silencing of LncSIK1 was critical to maintaining the progression of AML *in vivo*. (A‐G) NCG mice received subcutaneous injection with 5 × 10^6^ indicated NB4 cells. (A, B and C) Two weeks after the injection, the mice were sacrificed and the tumours were removed. The tumours were photographed in (B), and the weight was analysed in (C). The overexpression efficiencies of LncSIK1 in tumours were confirmed by qPCR and are shown in (A). (D, E and F) Protein expression of PCNA and CDK2 in tumours is shown in (D) and quantified in (E, PCNA) and (F, CDK2). (G) H&E staining in tumour tissues from mice that received NB4 cells. (H‐J) NCG mice received intravenous injection with 5 × 10^6^ indicated Molm13 cells. Mice that received PBS injections were the negative controls. (H) Three weeks after the injection, the luciferase signal intensities and locations of Molm13 cells in mice were analysed by bioluminescence imaging. (I) H&E staining of tissue slides of the bone marrow, liver, kidney and spleen from mice. (J) The proportion of human CD33^+^ cells in the peripheral blood of mice, as detected by flow cytometry. WT: cells infected with lentiviral LncSIK1‐WT plasmids. EV: cells infected with lentiviral empty vector plasmids. β‐actin served as a loading control. Three independent technical replicates (from three biological replicates) were performed and included in the analysis. *p < 0.05; **p < 0.01; ***p < 0.001. Bar, 50 μm

To explore the effect of LncSIK1 on tumour growth, we established a subcutaneous tumour model using NB4 cells. NCG mice received subcutaneous injections of selected NB4 cells (5 × 10^6^ each). The procedure is shown in Figure S1A. Two weeks after the injection, we killed the mice and assessed the tumour burden, the morphology of tumour tissue, and the expression of PCNA and CDK2 in the tumour. LncSIK1 was significantly enhanced by LncSIK1‐WT infection in the tumour (Figure 4A). Mice injected with LncSIK1‐WT‐NB4 cells appeared to carry smaller tumour burdens (Figure 4B). Compared with those from EV‐infected mice, the weight of tumours from LncSIK1‐WT‐infected mice was substantially lower (Figure 4C). In addition, the expression of PCNA and CDK2 was decreased by LncSIK1 in the tumour (Figure 4D, E and F). Indeed, the tumour tissue by haematoxylin and eosin (H&E) staining revealed that LncSIK1‐rescued tumours exhibited vacuole degeneration, cell nucleus shrinkage and edge collection, indicating severe damage to tumour cells (Figure 4G). Collectively, LncSIK1 arrested the growth of AML tumours *in vivo*.

To gain *in vivo* evidence that LncSIK1 can retard AML progression, we established a systemic model using Molm13 cells. We transplanted 5 × 10^6^ selected Molm13 cells into the tail vein of cyclophosphamide‐pretreated M‐NSG mice. The procedure is shown in Figure S1B. Three weeks after injection, AML progress indicated by organ infiltration was assessed using bioluminescence imaging (BLI), flow cytometry and H&E staining. Positive BLI signals were visible in the chest cavity, abdominal cavity and lower extremities (Figure 4H). Mice that received LncSIK1‐WT‐infected cells displayed a reduced signal intensity compared with EV cells, indicating a reduced progression of AML. H&E staining of the internal organs (liver, spleen, kidney, and lower extremities) showed mild infiltration of LncSIK1‐WT‐Molm13 cells and strong infiltration of EV‐Molm13 cells (Figure 4I). The results observed from flow cytometry also showed that the survival of circulating Molm13 cells was prominently reduced by LncSIK1, as demonstrated by the declined percentages of CD33‐positive cells in the peripheral blood (Figure 4J). Collectively, LncSIK1 impeded the progression of AML *in vivo*.

In summary, rescue of LncSIK1 helped to arrest AML development *in vivo*.

### 3.4 LncSIK1 was expressed at low levels in HSPCs and upregulated during G‐CSF‐induced differentiation of HSPCs in vitro

**FIGURE 5:**
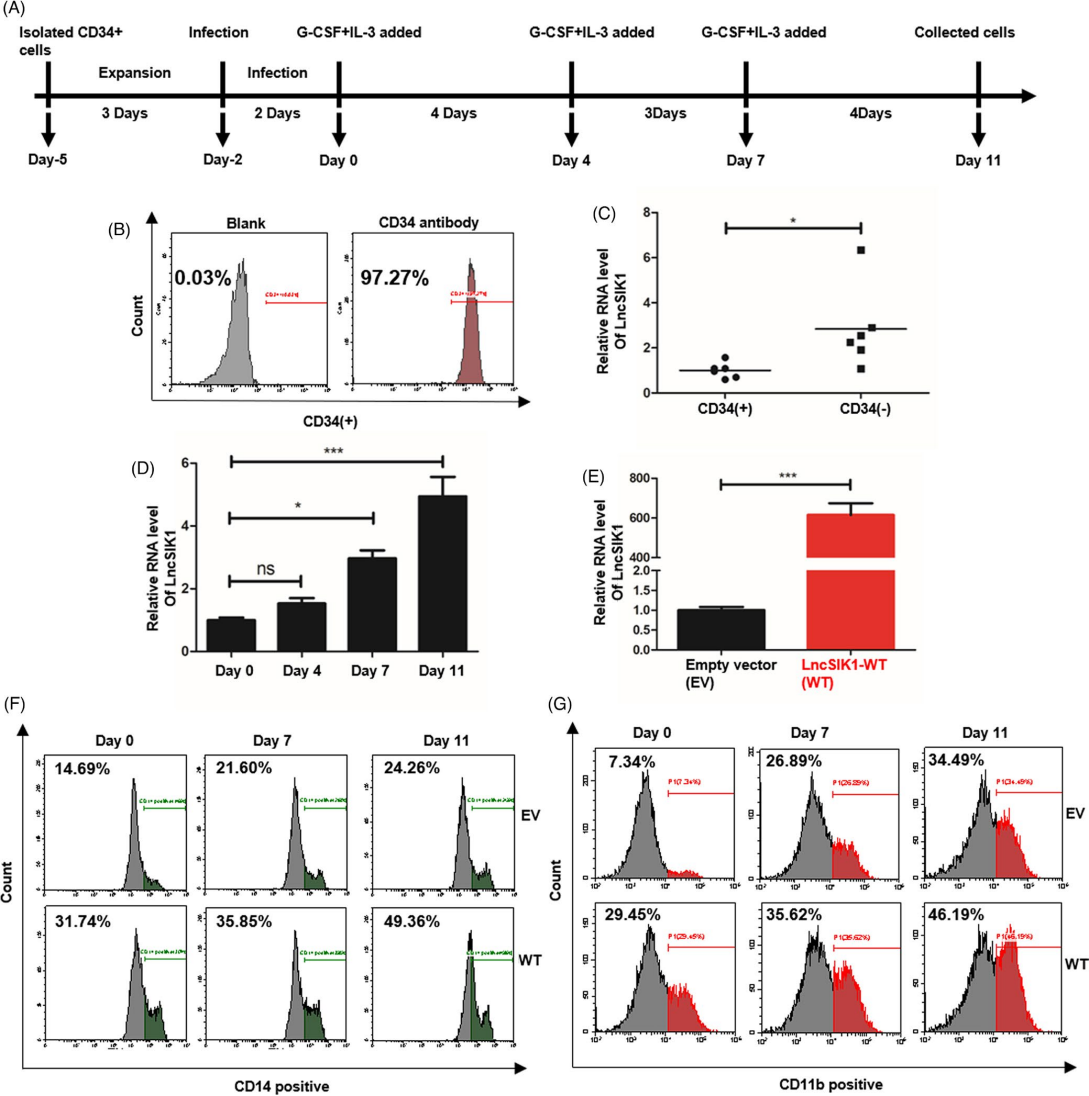
LncSIK1 was expressed at low levels in HSPCs and upregulated during G‐CSF‐induced differentiation of HSPCs *in vitro*. (A) The procedure of inducing human CD34^+^ HSPCs towards monocyte/granulocyte differentiation is shown in a schematic diagram. Note that the day when G‐CSF and IL‐3 were first added to induce maturation was set as Day 0. (B) The purity of CD34^+^ cells after magnetic separation was analysed by flow cytometry. (C) Relative expression of LncSIK1 in CD34^+^ and CD34^−^ cells, as measured by qPCR. (D) qPCR showing changes in LncSIK1 during G‐CSF+IL‐3‐induced differentiation in CD34^+^ cells. (E, F and G) Effects of LncSIK1 enhancement on the differentiation of CD34^+^ cells. CD34^+^ cells were infected with lentiviral LncSIK1‐WT (WT) or empty vector (EV) plasmids and treated with G‐CSF and IL‐3. The overexpression efficiency of LncSIK1‐WT in CD34^+^ cells was confirmed by qPCR and is shown in (E). *In vitro* induced human CD34^+^ cells were stained with CD14 (F) and CD11b (G) antibodies at the indicated time points and analysed by flow cytometry. WT: cells infected with lentiviral LncSIK1‐WT plasmids. EV: cells infected with lentiviral empty vector plasmids. β‐actin served as a loading control. Three independent technical replicates (from three biological replicates) were performed and included in the analysis. *p < 0.05; **p < 0.01; ***p < 0.001

CD34^+^ HSPCs sorted from human cord blood for *in vitro* differentiation assays were used to investigate whether LnsSIK1 could accelerate normal myelopoiesis. The procedure is shown in Figure 5A,^20^ and the efficiency of CD34 sorting was analysed by flow cytometry (Figure 5B). First, a prominently higher level of LncSIK1 in the CD34^−^ proportion was observed compared to the corresponding CD34^+^ proportion counterpart in MNCs (Figure 5C). Subsequently, CD34^+^ HSPCs were induced towards myelopoiesis by treatment with G‐CSF and IL‐3.^21^ Examinations revealed that LncSIK1 was gradually upregulated during myelopoiesis (Figure 5D). We next forced LncSIK1 expression in human CD34^+^ HSPCs by infection with lentiviral LncSIK1‐WT plasmids (Figure 5E). Notably, LncSIK1‐enhanced HSPCs displayed an advantage in myeloid maturation. An acceleration in differentiation was observed upon LncSIK1 enhancement, as measured by the levels of the differentiation surface markers, CD14 (Figure 5F) and CD11b (Figure 5G).

These results suggested that LncSIK1 accelerated G‐CSF‐induced differentiation of HSPCs *in vitro*.

### 3.5 LncSIK1 regulated the transcription of LC3 and DRAM, which might be modulated by E2F1

**FIGURE 6:**
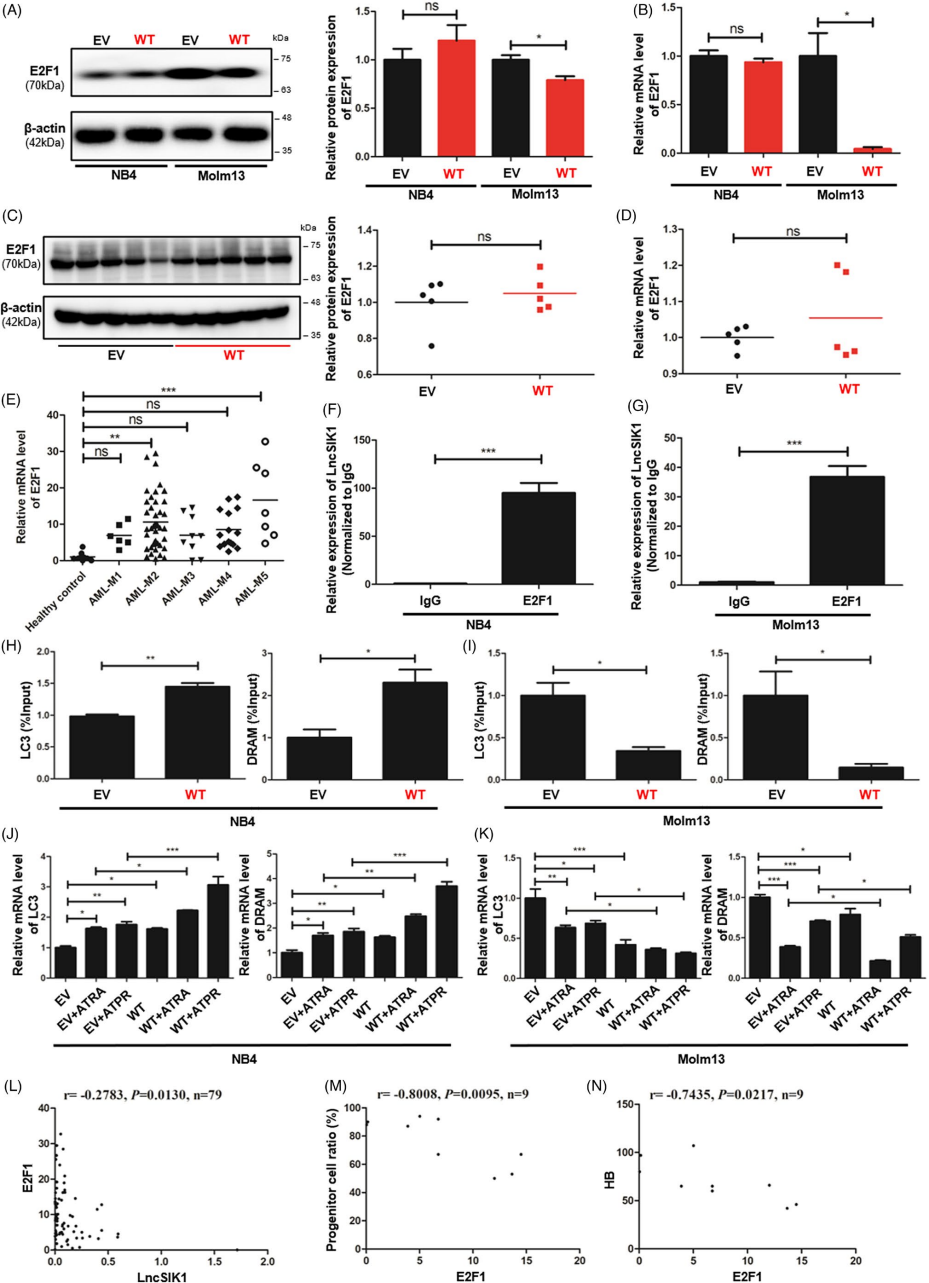
LncSIK1 regulated the transcription of LC3 and DRAM, which might be modulated by E2F1. (A and B) Effect of LncSIK1 enhancement on E2F1 in NB4 and Molm13 cells. Protein expression was detected by Western blot, represented on the left (A) and quantified on the right (A). The relative mRNA level of E2F1 was detected by qPCR and quantified in (B). (C and D) Effect of LncSIK1 enhancement on E2F1 in subcutaneous NB4 tumours. Protein expression was detected by Western blot, represented on the left (C) and quantified on the right (C). The relative mRNA level of E2F1 was detected by qPCR and quantified in (D). (E) Relative expression of E2F1 in BMNCs from AML primary patients (AML M1‐M5 subtypes) and BMNCs from healthy donors (control), as detected by qPCR. AML‐M1 (*N* = 6), AML‐M2 (*N* = 42), AML‐M3 (*N* = 9), AML‐M4 (*N* = 15), AML‐M5 (*N* = 7) and healthy controls (*N* = 10). Error bars depict mean ± SEM. (F and G) RIP assays were carried out to determine the enrichment of LncSIK1 in the E2F1 complex in NB4 (F) and Molm13 (G) cells. The relative quantities of LncSIK1 were analysed by qPCR and normalized to the IgG groups. (H and I) DNA ChIP assays were applied to measure E2F1 enrichment at the E2F1‐binding DNA regions in the promoter of LC3 or DRAM in NB4 (H) and Molm13 (I) cells. The relative quantities of LncSIK1 were analysed by qPCR and normalized to the input groups. (J and K) Effects of LncSIK1 enhancement on the transcription of LC3 and DRAM in NB4 (J) and Molm13 (K) cells, as detected by qPCR. (L) Pearson's correlation analysis of E2F1 expression and LncSIK1 in BMNCs from AML patients. (M and N) Pearson's correlation analysis of E2F1 expression and progenitor cell ratio (M) or amount of peripheral blood haemoglobin (HB, N) in AML patients. WT: cells infected with lentiviral LncSIK1‐WT plasmids. EV: cells infected with lentiviral empty vector plasmids. β‐actin served as a loading control. IgG served as a negative control. Three independent technical replicates (from three biological replicates) were performed and included in the analysis. ns: no significance, *p < 0.05; **p < 0.01; ***p < 0.001

We next sought to uncover the molecular mechanisms underlying LncSIK1. In our previous study, we found that LncSIK1 was implicated in the pathways regulated by E2F1.^8^ However, only minor changes in the protein (Figure 6A) and mRNA (Figure 6B) levels of E2F1 were observed in NB4 cells following LncSIK1 enhancement in this study. Similar results were found in NB4 tumours, in which LncSIK1 enhancement exerted a minor effect on E2F1 at the protein (Figure 6C) and mRNA levels (Figure 6D) during tumour growth. In contrast, the protein and mRNA levels of E2F1 were both inhibited by LncSIK1 in Molm13 cells (Figure 6A and B), suggesting a specific relationship between LncSIK1 and E2F1 in different AML subtypes. Subsequently, the LncSIK1‐E2F1 interaction was confirmed by the RIP‐qPCR assay, and the results showed that an E2F1 antibody can precipitate endogenous LncSIK1 in NB4 (Figure 6F) and Molm13 (Figure 6G) cells, encouraging us to further explore the downstream pathway of the LncSIK1/E2F1 axis. In addition to being a fundamental regulator of the cell cycle, E2F1 is reported to modulate the transcription of the autophagy genes LC3 and DRAM.^22^ Moreover, our previous KEGG analysis showed that lncRNAs upregulated by ATPR were primarily enriched in the lysosomal pathway.^8^ Therefore, a ChIP‐qPCR assay with the E2F1 antibody was performed to test the hypothesis that LncSIK1 is involved in autophagy by interacting with E2F1 and regulating E2F1‐dependent transcription of LC3 and DRAM. Compared to the nonspecific control (IgG), the DNA fragments of the LC3 or DRAM promoter containing the E2F1‐binding site were significantly enriched in the chromatin that was precipitated with anti‐E2F1 antibodies. Notably, enhanced expression of LncSIK1 strengthened E2F1 enrichment at the promoters in NB4 cells (Figure 6H) and surprisingly decreased E2F1 enrichment at the promoters in Molm13 cells (Figure 6I). Consistent results were observed at the mRNA levels of LC3 and DRAM, which were transcriptionally enhanced by LncSIK1 in NB4 cells (Figure 6J) and inhibited by LncSIK1 in Molm13 cells (Figure 6K). Furthermore, we found that E2F1 was expressed at a higher level in AML‐M2 and AML‐M5 patients (Molm13 cells represent the AML‐M5 subtype) than in healthy donors (Figure 6E). The results obtained from Pearson's correlation analysis suggested that only a weak negative correlation between LncSIK1 and E2F1 was observed in AML patients (*r* = −0.2783, *p* = 0.0130, *n* = 79; Figure 6L). These results are consistent with reported studies, in which E2F1 was demonstrated to be suppressed by inhibition of the FLT3 or STAT5 signalling pathway in Molm13 cells.^23^ Hence, the detailed network between LncSIK1 and E2F1 in AML needs further investigation. We subsequently analysed the correlation between E2F1 and AML. Despite the unsatisfactory amounts of specimens, in AML‐M3 patients, the proportion of progenitor cells in BMNCs (immunophenotyping) was negatively correlated with E2F1 expression (r=−0.8008, *p *= 0.0095, *n* = 9; Figure 6M), and the haemoglobin (HB) level was also negatively correlated with E2F1 expression (*r* = −0.7435, *p *= 0.0217, *n* = 9; Figure 6N). These results also indicated a complex role of E2F1 in the prognosis of AML patients.

These data showed that E2F1‐mediated transcription of LC3 and DRAM could be found in the LncSIK1 regulatory network in AML cells.

### 3.6 Autophagy was involved in LncSIK1‐mediated cellular development in AML

**FIGURE 7:**
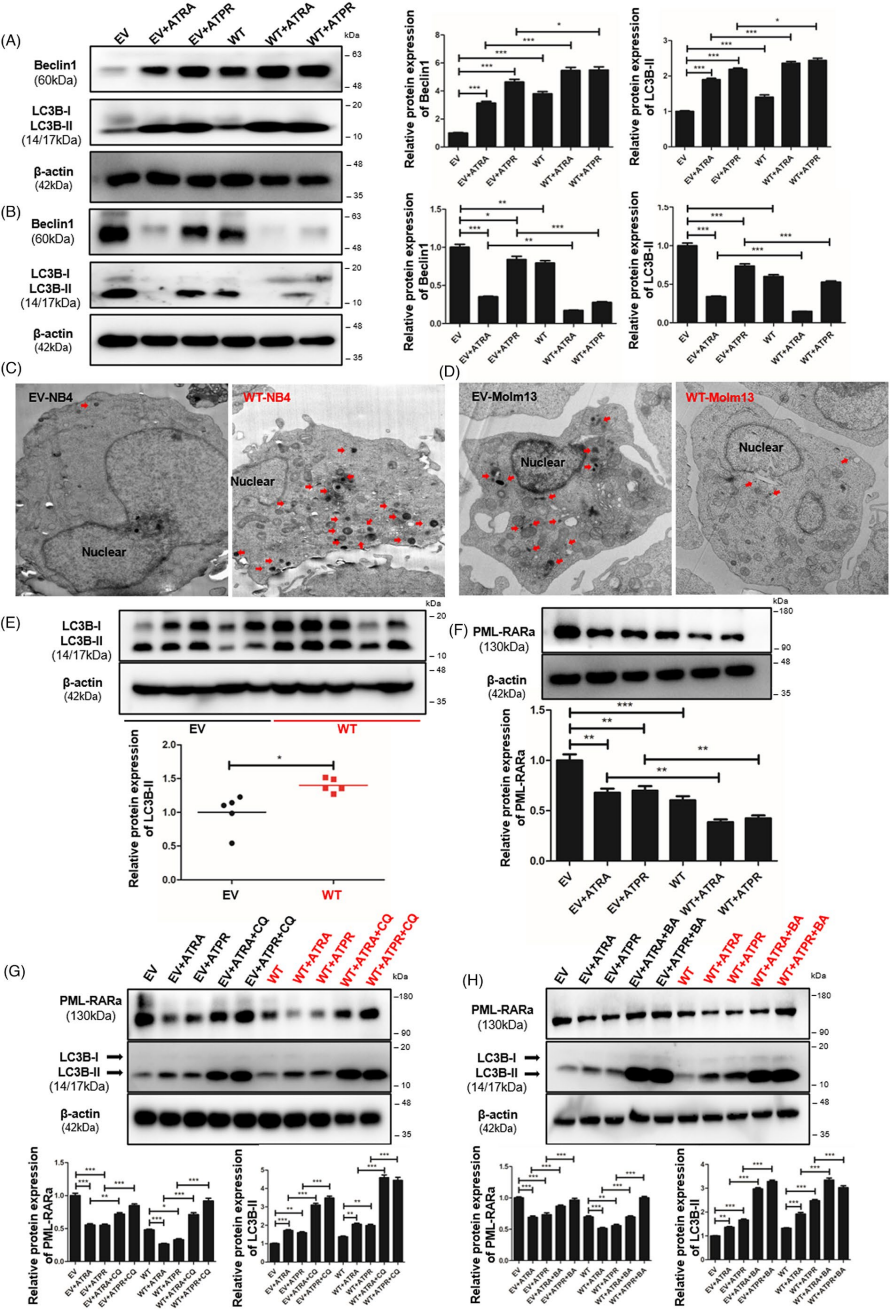
Autophagy was involved in LncSIK1‐mediated cellular development in AML. (A and B) Western blot analysis of the autophagy markers LC3B and Beclin1 in NB4 (A) and Molm13 (B) cells. (C and D) Transmission electron microscopy images of autophagosomes and autophagolysosomes in NB4 (C) and Molm13 (D) cells. Arrows represent autophagosomes and autophagolysosomes. (E) Western blotting was performed to detect the protein levels of the autophagy markers LC3B and Beclin1 in subcutaneous NB4 tumours. (F) Effect of LncSIK1 enhancement on the protein level of PML‐RARa in NB4 cells, as detected by Western blot. (G and H) Effect of autophagy inhibitor chloroquine (CQ, 40 μM, 6 h; G) or Bafilomycin A1 (BA, 25 nM, 12 h; H) on LncSIK1‐mediated clearance of PML‐RARa in NB4 cells. WT: cells infected with lentiviral LncSIK1‐WT plasmids. EV: cells infected with lentiviral empty vector plasmids. β‐actin served as a loading control. Three independent technical replicates (from three biological replicates) were performed and included in the analysis. ns: no significance, *p < 0.05; **p < 0.01; ***p < 0.001

Given the interplay between E2F1‐autophagy and LncSIK1, we hypothesized that LncSIK1 might be regulated through an autophagy pathway. In NB4 cells, rescue of LncSIK1 activated autophagy in the absence or presence of retinoic acid, as characterized by the elevation of LC3B cleavage and turnover (LC3B‐II), Beclin1 (Figure 7A), and the formation of autophagosomes (Figure 7C). In addition, LC3B‐II was also elevated by LncSIK1 in NB4 tumours (Figure 7E). However, the expression of LC3B‐II and Beclin1 (Figure 7B) and the formation of autophagosomes (Figure 7D) were blocked by LncSIK1 in Molm13 cells. These results were consistent with those observed in LncSIK1‐ and E2F1‐regulated AML progression, stressing once more the heterogeneity and complexity of AML. Many genetic mutants in AML have been shown to have prognostic importance. Patients with AML‐M3 carry a specific t (15;17) chromosomal translocation, resulting in the oncoprotein PML‐RARa. To examine the molecular signature governing leukaemia maintenance driven by LncSIK1 loss, we asked the role of LncSIK1 in PML‐RARa catabolism. We found that LncSIK1 enhanced the clearance of PML‐RARa in the absence or presence of retinoic acid (Figure 7F). To directly assess whether autophagy is involved in the clearance of PML‐RARa regulated by LncSIK1, we used bafilomycin A1 (BA), a lysosomal proton pump inhibitor, and chloroquine (CQ), a lysosomotropic agent, on NB4 cells to block autophagic‐lysosomal degradation by decreasing autophagic flux formation.^24^, ^25^ As expected, we noticed a clear accumulation of PML‐RARa after BA (Figure 7G) or CQ treatment (Figure 7H) in NB4 cells, especially in LncSIK1‐rescued NB4 cells. These data suggested that low levels of LncSIK1 in NB4 cells may help to maintain PML‐RARa retention. From this, it is clear that we observed a correlation between LncSIK1/autophagy and therapy‐induced clearance of PML‐RARa or differentiation in NB4 cells.

In conclusion, LncSIK1 induced autophagy in NB4 cells to clear the PML‐RARa oncoprotein, in contrast, the high basal autophagy level in Molm13 cells was reversed by LncSIK1.

## 4 DISCUSSION

In the current study, we found that the silencing of LncSIK1 was critical for maintaining AML, moreover, LncSIK1 enhanced the effect of retinoic acid on AML cells. In terms of mechanism, LncSIK1 rescued autophagy by interacting with E2F1 and recruiting E2F1 to bind with the promoters of LC3 and DRAM in NB4 cells. However, the interaction between LncSIK1 and E2F1 inhibited E2F1 levels and thus suppressed autophagy in Molm13 cells. This different role of autophagy is consistent with published studies, indicating that the role of the autophagy pathway in AML remains a topic of debate and controversy. Whether the lncRNA/E2F1 axis forms a distinctive autophagic regulatory network that plays multiple roles in AML progression is a question we need to consider.

In general, the heterogeneity caused by different genetic mutations leads to diverse roles of autophagy in AML. For example, researchers pointed out that AML1‐ETO‐positive patients may benefit from autophagy inhibitors.^26^ However, diminished autophagy accelerated the death of MLL‐ENL‐driven AML models, and downregulation of autophagy in mice transplanted with OCI‐AML3 cells improved overall survival after chemotherapy.^27^, ^28^, ^29^ Hence, strategies involving autophagy for AML cures still require deep assessment. The mechanisms underlying the diversity and whether it involves the lncRNA/E2F1 axis also need to be explored. Autophagy may be a help in alleviating AML patients with TP53 mutations due to its critical effect on mediating autophagic elimination of TP53.^30^ However, pharmacological blockage of autophagy restrains AMLs harbouring wild‐type P53.^31^ From an association study, LncMEG3 was strongly associated with AML leukaemogenesis via P53‐dependent pathways.^32^ Interestingly, LncMEG3 inhibits autophagy in lung cancer chemotherapy while inducing autophagy in nasopharyngeal carcinoma cells,^33^, ^34^ and the E2F1‐P53 regulatory axis suppresses replicative stress in differentiating cells and prevents tumorigenesis,^35^ establishing a potential effect of LncMEG3/E2F1/autophagy in regulating AML. In addition, as previously discussed, FLT3 activation by ITD mutation stimulates autophagic flux; however, it is apparently contradictory to the fact that autophagy mediates degradation of FLT3‐ITD oncogenic protein.^36^ It is worth discussing the regulatory axis of lncRNA/E2F1 in this contradictory network. Supporting evidence has shown that several lncRNAs are related to FLT3‐ITD, including LncH19,^37^ LncSOCS2‐AS,^38^ LncTUG1^39^ and LncCCAT2.^40^ Among them, LncH19, LncTUG1 and LncCCAT2 were implicated in autophagy and E2F1. Data are still limited, but the findings provide encouraging support for the hypothesis that the pros and cons of autophagy‐targeted therapies in AML may depend on the lncRNA/E2F1 axis. We believe that further investigation is useful for new strategies in AML treatment.

In addition to the discovery of lncRNAs, microRNAs (miRNAs) are also established regulators of lncRNA‐mediated transcription.^41^, ^42^ miRNAs are small RNAs of 22 nucleotides in length that can bind to complementary sequences in target transcripts and guide their degradation or prevent their translation. lncRNAs act as competitive endogenous RNAs (ceRNAs) to attenuate miRNA‐mediated activity and rescue target transcripts.^43^ Our data support future investigation on the underlying mechanism of LncSIK1 in the E2F1 regulatory network. We wondered whether the LncSIK1‐miRNA loop is responsible for the contradictory results of the LncSIK1‐E2F1 axis in different types of AML. Based on the cross‐regulated loop of ceRNA, we were interested in potential miRNAs related to the LncSIK1‐E2F1 pathway. Using miRDB,^44^ together with searching the literature, we preliminarily predicted the potential miRNAs, and the potential binding sites of LncSIK1 on various miRNAs are shown in Additional file S2. Hsa‐miR‐5002‐3p, hsa‐miR‐6833‐3p, hsa‐miR‐6873‐3p and hsa‐miR‐574‐5p are predicted to be potential miRNAs targeting LncSIK1. Although no direct relationship has been confirmed, the opposite expression of miR‐574‐5p and E2F1 was observed in senescent human fibroblasts, suggesting a hypothetical connection between miR‐574‐5p and E2F1.^45^ In addition, miR‐574‐5p may be implicated in childhood T‐cell acute lymphoblastic leukaemia.^46^ However, the LncSIK1/miR‐574‐5p/E2F1 axis needs further exploration. It is worth noting that the majority of predicted miRNAs show a close relation with malignancies, which supports that LncSIK1 is a conceivable cancer‐related gene.^47^, ^48^, ^49^ In view of the important role of miRNA in regulating autophagy, studies in LncSIK1/miRNA/E2F1/autophagy may benefit understanding the contradictory results.

In summary, our study concluded that LncSIK1 is silenced during AML leukaemogenesis and that rescue of LncSIK1 retards AML progression. Schematic and hypothetical regulatory networks are represented in the graphical abstract image and provide a potential regulatory mechanism for LncSIK1 in AML.

## CONFLICTS OF INTEREST

The authors declare no conflict of interest.

## AUTHOR CONTRIBUTIONS

K.W. and F.C. performed study concept and design; K.W., J.L. and G.D. performed the development of methodology and writing, review and revision of the paper; Z.O., S.L., and X.X. provided acquisition, analysis and interpretation of data, and statistical analysis; M.Z., and X.P. provided technical and material support. All authors read and approved the final paper.

## Supporting information

Fig S1

Supplementary Material

Supplementary Material

Supplementary Material

Supplementary Material

## Data Availability

The data sets used and/or analysed during the current study are available from the corresponding author on reasonable request.
