# Supplementary figures and images for: LncSIK1 enhanced the sensitivity of AML cells to retinoic acid by the E2F1/autophagy pathway
Source: Cell Prolif. 2022 Jan 29;55(3):e13185. doi: 10.1111/cpr.13185 (PMC8891555; doi:10.1111/cpr.13185)

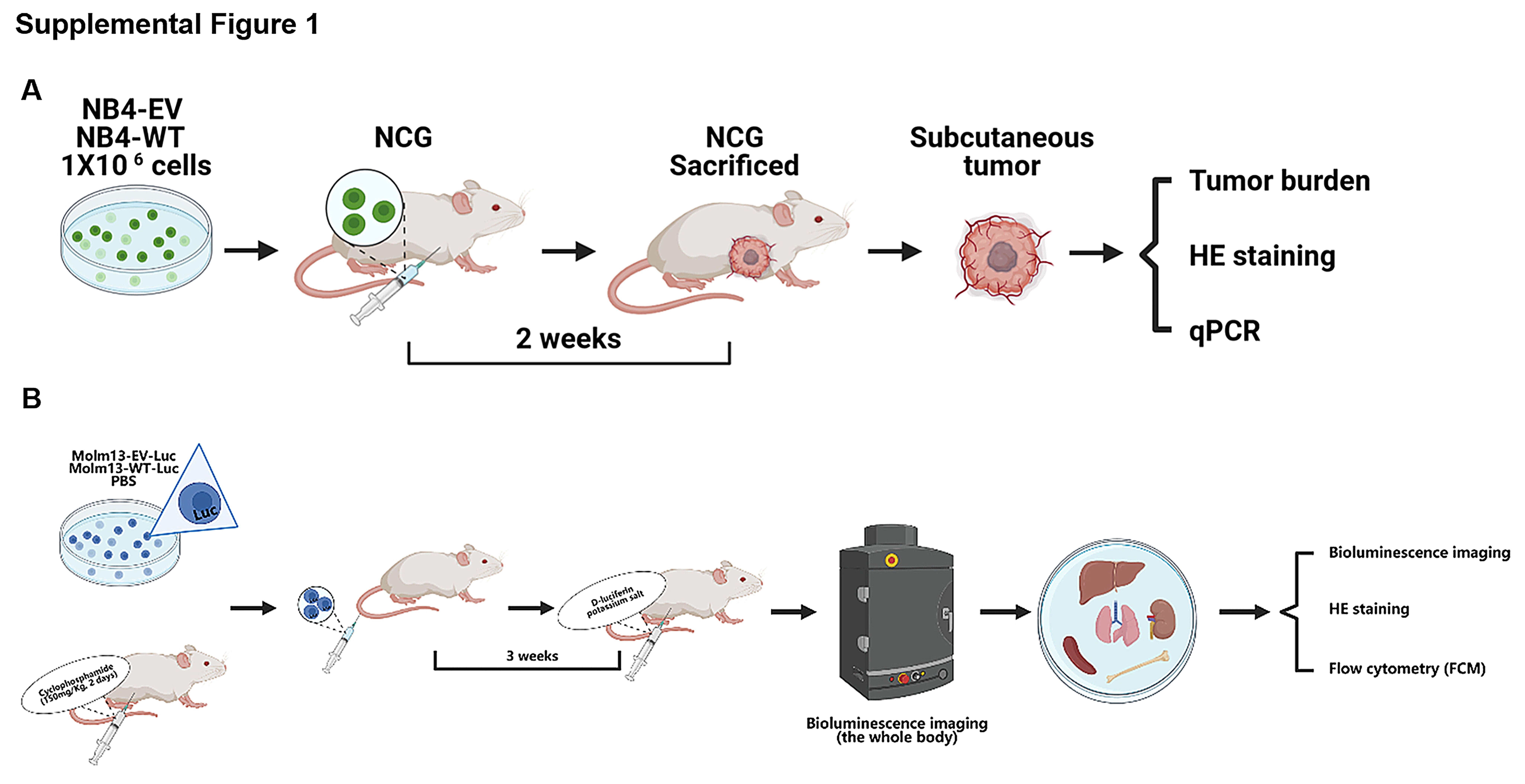

Supplement: Supplementary file 1 — Fig S1 [file CPR-55-e13185-s002.jpg]
