# Supplementary material for: LncSIK1 enhanced the sensitivity of AML cells to retinoic acid by the E2F1/autophagy pathway
Source: Cell Prolif. 2022 Jan 29;55(3):e13185. doi: 10.1111/cpr.13185 (PMC8891555; doi:10.1111/cpr.13185)
Supplement: Supplementary file 2 — Supplementary Material [file CPR-55-e13185-s001.docx]

**Additional file 1**

**Materials and Methods**

**1. Cell lines and cell culture**

NB4 cell line was purchased from Genechem (Shanghai, China). MV4-11 cell line was obtained from Procell Life Science&Technology (Wuhan, China). HL60 and THP-1 cell lines were purchased from Gecko gene (Shanghai, China). Molm13 cell line was donated by the University of Maryland School of Medicine (USA). All cells were maintained in regular RPMI-1640 medium (HyClone, USA) supplemented with 10% FBS (Biological industries, Israel) and 1% penicillin-streptomycin (Beyotime Biotechnology, China). All cells were cultured at 37 °C in a 5% CO^2^ atmosphere. All cell lines have passaged for fewer than 6 months on our laboratory after resuscitation.

**2.** **Xenotransplantation experiments**

NOD/ShiLtJGpt-Prkdc^em26Cd52^Il2rg^em26Cd22^/Gpt (NCG) immunodeficient mice (Animal certificate number: 202011417) were obtained from the Gempharmatech (Nanjing, China), NOD-Prkdc^scid^Il2rg^em1^/Smoc (M-NSG) immunodeficient mice (Animal certificate number: 20170010009479) were obtained from the Shanghai Model Organisms Center (Shanghai, China). Male mice at the age of 6-9 weeks were used for the experiments. All animals were fed under specific pathogen-free facility (Temperature:23-24°C, humidity: 40%-45%) in the Laboratory Animal Center of Anhui Medical University. All experiments on animals were performed in accordance with the institutional ethical guidelines for animal experiments (Ethics Number: LISC20190751).

For the subcutaneous tumor xenografts study procedure (Supplemental Figure 1A), selected LncSIK1-WT or EV NB4 cells diluted to a concentration of 5 × 10^7^ cells/mL in phosphate buffer saline (PBS) (HyClone, USA). NCG mice were subcutaneously injected with 0.1ml of the suspension (5 × 10^6^ cells) into either side of flank area. Mice were sacrificed 2 weeks later, and tumors were weighed, fixed and subjected to Hematoxylin and eosin (H&E) staining.

For the intravenous bone marrow engraftment study procedure (Supplemental Figure 1B), M-NSG mice were acclimated for 2 weeks before pretreatment with 150 mg/kg cyclophosphamide delivered intraperitoneally once a day for 2 days. After a 24-hour rest period, selected LncSIK1-WT or EV Molm13 cells were diluted to a concentration of 5 × 10^7^ cells/mL in PBS and M-NSG mice were injected with 0.1 ml of the suspension (5 × 10^6^ cells) into tail vein. 3 weeks after inoculation, the mice were sacrificed for analysis. Bioluminescence imaging (BLI) was applied to evaluate human cell infiltration (BLI positive signal) in mice. H&E staining and flow cytometry were employed to detect cell engraftment in bone marrow, peripheral blood, liver, kidney, and spleen as described.

**3. Human samples and cell isolation**

Primary AML leukemia cells were isolated from the bone marrow of AML patients at diagnose from the Second Hospital of Anhui Medical University. Briefly, bone marrow blood was slowly layered over Ficoll-Paque PLUS solution (GE Healthcare Life Sciences, Sweden) and centrifuged at 550 g for 25 minutes. The mononuclear cells in the interphase layer were carefully transferred into another fresh tube and washed with Hank’s balanced salt solution (Beyotime Biotechnology, China) twice and then applied to subsequent experiments. CD34^+^ hematopoietic stem/progenitor cells (HSPCs), and CD34^-^ cells were purified from cord blood of healthy donors from the First Affiliated Hospital of Anhui Medical University using Ficoll-Paque PLUS and anti-CD34-coated magnetic beads (Miltenyi Biotec, Germany). Briefly, we isolated the mononuclear cells from fresh cord blood as described above. Next, a single cell suspension obtained was incubated with anti-CD34-coated beads and selected on autoMACS Pro (Miltenyi Biotec, Germany). CD34^+^ cells were identified by incubating with CD34 antibody (BioLegend, USA) and analyzing with flow cytometry (CytoFLEX, Beckman Coulter, USA).

**4. Culture and induced differentiation of HSPCs**

Primary CD34^+^ cells were cultured in StemSpan SFEM medium (StemCell Technologies, Canada) supplemented with 2 mmol/L L-glutamine (Sigma-Aldrich, USA), 1% Lipid Mixture 1 (L0288, Sigma-Aldrich, USA), 100 ng/mL SCF (Human origin, PeproTech, USA), and 2 ng/mL IL-3 (Human origin, PeproTech, USA), 1% penicillin-streptomycin. To induce granulocyte differentiation, the cytokine cocktail of 50 μg/ml G-CSF (Human origin, PeproTech, USA) and 25 μg/ml IL-3 were freshly added to the culture media.

**5.** **Cell nucleus/cytoplasm fraction isolation**

The cell suspensions were centrifuged at 1, 200 rpm for 5 minutes at room temperature. The cell pellet was washed with cold PBS and then incubated with a low permeability solution (10 mM HEPES-NaOH, PH7.9; 10 mM KCL; 1.5 mM MgCL2; 4% cocktail) for 20 minutes. After hypotonic lysis, 8 μL NP-40 (5%) was added into the mixture. Then the mixture was allowed to vortex and fully lyse. Next, we centrifuged the mixture at 5, 000 g for 5 minutes, and then transferred the supernatant (Cytoplasm fraction) into a new tube. The precipitation (Nucleus fraction) was washed twice with cold PBS. Finally, the cell nucleus and cytoplasm fraction were applied to extract total RNA.

**6. Protein extraction and western blotting**

Total proteins were extracted from cells using RIPA lysis buffer (Beyotime Biotechnology, China) with PMSF (Beyotime Biotechnology, China). Cells were collected, washed with cold PBS, and lysed with RIPA buffer. Cell lysates were centrifuged at 12, 000 rpm for 30 minutes. The supernatant was transferred, mixed with SDS-PAGE loading buffer, and separated by SDS-PAGE. Then, the separated protein was transferred onto methanol-activated PVDF membranes (Millipore, USA). The membranes were blocking with 5 % defatted milk for 2 hours at room temperature, and then incubated with the appropriate diluted antibodies against CDK2 (Abcam, United Kingdom), PCNA (Abcam, United Kingdom), PML-RARa (Cell signaling technology, USA), LC3 (Abcam, United Kingdom), Beclin1 (Cell signaling technology, USA), E2F1 (Cell signaling technology, USA), β-actin (Proteintech, USA), respectively, at least for 8 hours. After the incubation, membranes were hatched with HRP-conjugated secondary antibody (ZSGB-Bio, China). The blots were visualized with Omni-ECL™Femto Light Chemiluminescence Kit (EpiZyme, China) and quantified with ImageJ software.

**7. Bioluminescence imaging (BLI)**

Mice were depilated and received intraperitoneal injection with D-Luciferin potassium salt (150mg/kg, Abcam, United Kingdom). After 20-30 minutes, leukemia cell signals *in vivo* were monitored by bioluminescence imaging using the SPECTRAL ami HTX (USA).

**8.** **Total RNA extraction and quantitative real-time PCR analysis (qPCR)**

Total RNA was extracted from cells using Trizol procedure (Invitrogen, USA). RNA was reverse transcribed into cDNA with the reverse transcription system (Accurate Biology, China). The primers used for qPCR were as Additional file 3. All primers were synthesized by Sangon Biotech (Shanghai, China) or General BIOL (Anhui, China). All of the data were normalized to β-actin as an internal control.

**9. Flow cytometry for cell differentiation analysis**

The proportion of monocytes/macrophage (CD14-positive), granulocytes (CD11b-positive or CD14-positive), and HSPCs (CD34-positive) were measured by flow cytometry. In brief, cells with different treatments were collected, washed with ice-cold PBS, and incubated with the specific antibodies with fluorescence labeling of differentiation markers, CD14-FITC and CD11b-PE (BioLegend, USA) for 30 minutes in dark at room temperature. Finally, the cells were washed twice with PBS and then analyzed on a flow cytometry.

**10.** **Morphological assessment**

Cell morphology was detected by Wright-Giemsa Staining, which staining the nucleus into purple and the cytoplasm into blue. In short, the cells were harvested and resuspended with the culture medium. Then cells were uniformly coated on a glass slide and stained with Wright-Giemsa Stain Solution. Finally, the slides were observed under a Fluorescence Inversion Microscope System (OLYMPUS, Japan).

**11. Cell transfection**

LncSIK1 enhancement was achieved through lentiviral infection of pHBLV-CMV-LncSIK1-EF1-fLUC-T2A-PURO plasmids. Lentiviral carrying LncSIK1-WT or empty vectors plasmid was obtained from Hanbio (Shanghai, China) and infections were performed according to the manufacturer’s manual. Stable infected cells were selected by puromycin. The selected cells were cultured in a medium containing 2 μg/ml puromycin. qPCR was employed to confirm the enhancement of LncSIK1 in cells.

**12. Fluorescence *in situ* Hybridization (FISH)**

The location of LncSIK1 in the cell was identified by Fluorescence *in situ* Hybridization. LncSIK1 and U6 probes were made by RiboBio (Guangzhou, China). U6 is used as a control for nucleus. Cells were uniformly coated on cationic anti-dropping slides. The hybridization procedure was performed as the manufacturer’s manuals. Fluorescence imaging was detected by a Fluorescence Inversion Microscope System.

**13. RNA immunoprecipitation assay (RIP)**

The interaction between E2F1 and LncSIK1 was determined by the RIP kit (BersinBio, China) in accordance with the manufacturer’s instructions. Total RNA from RIP product was extracted by RNA extraction mixture (Phenol-Chloroform-Isoamyl alcohol: 125-24-1). Qualitative analysis of the interaction was performed by qPCR.

**14. Chromatin immunoprecipitation assay (ChIP)**

The enrichment of E2F1 in the promoter of LC3 or DRAM was determined by ChIP kit (Beyotime Biotechnology, China) in accordance with the manufacturer’s instructions. The protein-DNA complexes were immunoprecipitated with anti-E2F1 antibodies or rabbit control IgG. The DNA fragments precipitated in the complexes was subjected to PCR amplification of the LC3 or DRAM promoter region using the primers listed in Additional file 3. Quantitative analysis of the total DNA products was performed by qPCR.

**15.** **Statistical analysis**

Data were assessed using the software SPSS 19.0 (SPSS, USA). Data with a normal distribution were analyzed by Student’s t-test, while data with a nonnormal distribution were analyzed by the Mann-Whitney nonparametric test. Comparisons between groups were performed using one-way ANOVA followed by Tukey’s multiple comparison. Correlations between the samples were evaluated statistically through Pearson’s correlation coefficient. Error bars depict the mean ± standard error of the mean (SEM). P values of less than .05 were considered as statistically significant.
