# Supplementary material for: LncSIK1 enhanced the sensitivity of AML cells to retinoic acid by the E2F1/autophagy pathway
Source: Cell Prolif. 2022 Jan 29;55(3):e13185. doi: 10.1111/cpr.13185 (PMC8891555; doi:10.1111/cpr.13185)
Supplement: Supplementary file 3 — Supplementary Material [file CPR-55-e13185-s004.docx]

**Additional file 2**

**Predicted miRNA targets of LncSIK1**

**Has-miR-5002-3p**

Sequence: UGACUGCCUCACUGACCACUU

Target Score:94

**Has-miR-6833-3P**

Sequence: UUUCUCUCUCCACUUCCUCAG

Target Score:77

**Has-miR-6873-3p**

Sequence: UUCUCUCUGUCUUUCUCUCUCAG

Target Score:67

**Has-miR-574-5p**

Sequence: UGAGUGUGUGUGUGUGAGUGUGU

Target Score:63
