# Supplementary material for: LncSIK1 enhanced the sensitivity of AML cells to retinoic acid by the E2F1/autophagy pathway
Source: Cell Prolif. 2022 Jan 29;55(3):e13185. doi: 10.1111/cpr.13185 (PMC8891555; doi:10.1111/cpr.13185)
Supplement: Supplementary file 4 — Supplementary Material [file CPR-55-e13185-s005.docx]

**Additional file 3**

**Primers**

**For the gene encoding LncSIK1:**

5’ CTGCTTGCCCTAGAGAGAAA and 5’ TATCTGCTGCACCGAGTC

**For the gene encoding E2F1:**

5’ CGGCGATGTTACGACATTA and 5’ CTTGTGGTAGTCTAGTTCTTGC

**For the gene encoding LC3:**

5’ AGACCTTCAAGCAGCGCCG and 5’ ACACTGACAATTTCATCCCG

**For the gene encoding DRAM:**

5’ CCGCCTTCATTATCTCCTAC and 5’ CCCATTCCGAAACATCCCAC

**For the gene encoding PML-RARa:**

5’ CCGATGGCTTCGACGAGTTC and 5’ CTCACAGGCGCTGACCCCAT

**For the gene encoding LC3 promoter:**

5’ TGGAGGGGAAAGGATGGTCG and 5’ GGGGCGGAGCAGGTGTGTG

**For the gene encoding DRAM promoter:**

5’ GCTGATCTCTAGTTCTTGGG and 5’ ACACCCGAAAACCCAGACAG

**For the gene encoding U6:**

5’ CTCGCTTCGGCAGCACA and 5’ AACGCTTCACGAATTTGCGT

**For the gene encoding β-actin:**

5’ ACTCTTCCAGCCTTCCTTCC and 5’ TCCTTCTGCATCCTGTCAGC
