# Supplementary material for: LncSIK1 enhanced the sensitivity of AML cells to retinoic acid by the E2F1/autophagy pathway
Source: Cell Prolif. 2022 Jan 29;55(3):e13185. doi: 10.1111/cpr.13185 (PMC8891555; doi:10.1111/cpr.13185)
Supplement: Supplementary file 5 — Supplementary Material [file CPR-55-e13185-s003.docx]

**Supplemental Figure Legends**

**Supplemental Figure 1**

**(A)** Scheme of the process of generating AML subcutaneous xenograft.

**(B)** Scheme of the process of generating AML intravenous engraftment.
